# Potential bidirectional regulatory effects of botanical drug metabolites on tumors and cardiovascular diseases based on the PI3K/Akt/mTOR pathway

**DOI:** 10.3389/fphar.2025.1467894

**Published:** 2025-03-24

**Authors:** Su-ya Ma, Yong-mei Liu, Jie Wang

**Affiliations:** Guang’anmen Hospital, China Academy of Chinese Medicine Sciences, Beijing, China

**Keywords:** botanical drug metabolite, PI3K/AKT/mTOR, cancer, cardiotoxicity, cardioprotective

## Abstract

Pharmacological interventions targeting the phosphatidylinositol-3-kinase/protein kinase B/mammalian target of rapamycin (PI3K/Akt/mTOR) signaling pathway are predominantly employed as anticancer therapies, yet they are frequently associated with significant cardiac toxicity. Additionally, the PI3K/Akt/mTOR pathway plays a crucial role in the treatment of cardiovascular diseases, highlighting its dual significance in both oncology and cardiology. Therefore, the PI3K/Akt/mTOR pathway has become an ideal signaling pathway for studying cardioprotection, anticancer effects, and their associated cardiac toxicity. Botanical drugs have emerged as a significant source for developing therapeutic agents with anticancer and cardioprotective effects, often exhibiting bidirectional protective properties. Consequently, this study investigates the bidirectional regulatory influence of botanical drug metabolites in oncology and cardiology via the PI3K/Akt/mTOR pathway. The research indicated that the PI3K/Akt/mTOR signaling pathway plays a critical regulatory role in the pathogenesis of both tumors and cardiovascular diseases. The botanical drug metabolites Ruscogenin, Sulforaphane, Naringenin, Kaempferol, Poncirin, and Puerarin can improve cancer by inhibiting the phosphorylation levels within the PI3K/Akt/mTOR signaling cascade. Moreover, they also provide cardioprotective effects in cardiac injury conditions by activating the phosphorylation levels of the PI3K/Akt/mTOR pathway. Therefore, the phosphorylation dynamics of key components in the PI3K/Akt/mTOR pathway, particularly the phosphorylation of Akt, along with the functional implications of different phosphorylation sites, may offer new therapeutic strategies and insights for cancer treatment and the mitigation of cardiotoxicity associated with cancer therapies.

## 1 Introduction

The phosphatidylinositol-3-kinase/protein kinase B/mammalian target of rapamycin (PI3K/Akt/mTOR) signaling pathway is a key regulator of diverse physiological processes, including cell cycle progression, growth, and proliferation, through the activation of downstream effectors (Ghafouri-Fard et al., 2022). This pathway is implicated in the pathogenesis of various human diseases, particularly cardiovascular disorders, where it modulates cardiomyocyte size, survival, angiogenesis, and inflammatory responses. Conversely, aberrant activation of the PI3K/Akt/mTOR pathway has been identified as a critical driver in the initiation and progression of multiple cancers (He et al., 2021). Given its central role in both cardiovascular and oncological contexts, the PI3K/Akt/mTOR pathway represents a promising target for therapeutic intervention. However, existing anti-tumor agents targeting this pathway, such as Idelalisib, Everolimus, and Capivasertib, often exhibit significant cardiotoxicity. Thus, elucidating the distinct mechanisms of the PI3K/Akt/mTOR pathway in cardiovascular and tumor biology is essential for optimizing drug development and minimizing adverse effects. The development of novel compounds capable of simultaneously exerting cardioprotective and anti-tumor effects through this pathway represents an attractive and unmet clinical need.

**FIGURE 1:**
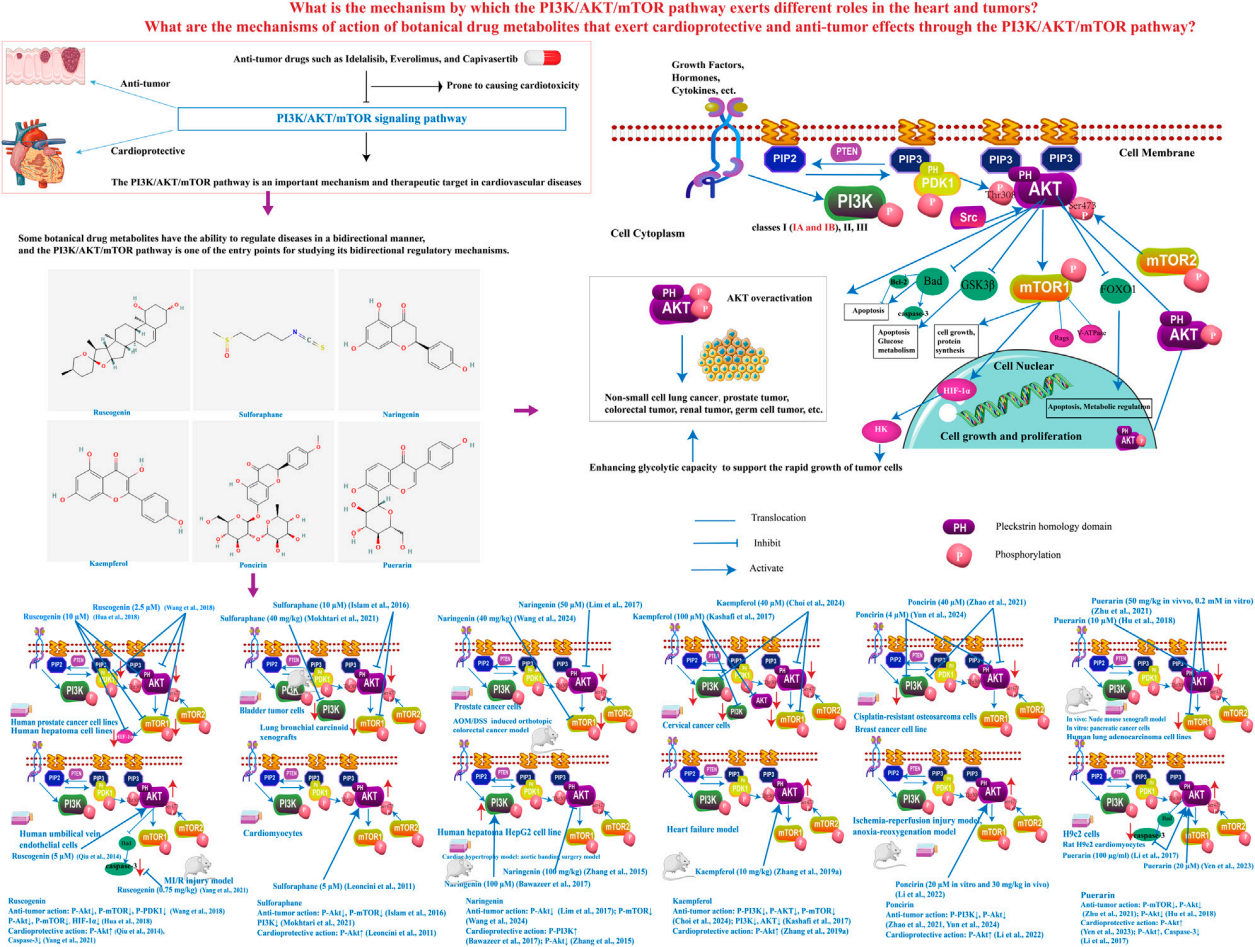
Investigation of bidirectional regulatory mechanisms of botanical drug metabolites targeting the PI3K/Akt/mTOR pathway. The PI3K/Akt/mTOR signaling pathway is activated by G protein-coupled receptors and receptor tyrosine kinases through ligands such as growth factors, hormones, or cytokines. These receptors recruit PI3K to the plasma membrane to catalyze PIP2 phosphorylation into PIP3, a process antagonized by PTEN-mediated dephosphorylation. PIP3 recruits PH domain-containing proteins (e.g., Akt and PDK1) to the membrane, where Akt, as the primary downstream effector, is fully activated by PDK1 (Thr308) and mTORC2 (Ser473). Activated Akt phosphorylates multiple targets: FOXO1 phosphorylation triggers cytoplasmic retention to suppress apoptosis and metabolic regulation; GSK3β phosphorylation inhibits its kinase activity, promoting glycogen synthesis and proliferation; Bad phosphorylation separates it from Bcl-2, inhibiting its pro-apoptotic activity and blocks cytochrome c release from mitochondria, inhibiting apoptosome-dependent Caspase-3 activation; and mTORC1 activation enhances protein synthesis and cell growth. mTORC1 stabilizes HIF-1α by inhibiting degradation, boosting its translation and transcriptional activity, thereby upregulating hexokinase to accelerate glycolysis for tumor survival. Hyperactivated Akt is clinically implicated in malignancies including NSCLC, prostate, colorectal, renal, and germ cell cancers. PDK1, phosphoinositide-dependent kinase 1; PIP2, phosphatidylinositol 4,5-bisphosphate; PIP3, phosphatidylinositol 3,4,5-trisphosphate; PTEN: phosphatase and tensin homolog; mTORC2, mechanistic target of rapamycin complex 2; mTORC1, mechanistic target of rapamycin complex 1; FOXO1, forkhead box protein O1; GSK3β, glycogen synthase kinase three beta; Bad, Bcl-2-associated death promoter.

Botanical drugs, renowned for their multifaceted attributes such as multi-component composition, multi-target biological activity, and minimal side effects (Liang et al., 2024), have long been treasured as a rich source of anticancer and cardioprotective agents. Multiple signaling pathways have been found to be influenced by botanical drugs via modulating the activity or expression of their molecular targets (Naeem et al., 2022). A variety of botanical drugs and its extracts have been found to have bidirectional regulatory effects on diseases. For instance, *Astragalus membranaceus* (*Fisch.*) *Bge.var.mongholicus* (*Bge.*) *Hsiao* (belong to the Fabaceae families, also named *Astragalus mongholicus Bunge*) has a bidirectional regulatory effect on blood pressure, *Panax notoginseng (Burk.) F. H. Chen* (belong to the Araliaceae families, also named *P. notoginseng (Burkill) F.H.Chen*) has the functions of promoting blood circulation and hemostasis (Peng et al., 2022). The mechanisms underlying these bidirectional regulatory effects in botanical drugs are intriguing and complex. The differences in the mechanisms of the PI3K/Akt/mTOR pathway in cardiovascular and tumor contexts provide an excellent entry point for this research. Therefore, this study aims to explore the bidirectional regulatory mechanisms of the PI3K/Akt/mTOR pathway in both cardiovascular diseases and tumors, focusing on botanical drug metabolites that exhibit such bidirectional effects. Investigating the potential bidirectional regulatory mechanisms of botanical drug metabolites in cancer and cardiovascular diseases is of significant importance for the development and mechanistic elucidation of botanical drugs (Figure 1).

## 2 Perspectives on the PI3K/Akt/mTOR signaling mechanism

The PI3K/Akt/mTOR signaling pathway is integral to a spectrum of cellular processes essential for life, including survival, growth, proliferation, angiogenesis, and metabolism (Su et al., 2023). PI3Ks constitute a diverse family of lipid kinases, stratified into three distinct classes—Class I, II, and III—based on their structural attributes and regulatory mechanisms within the signaling pathway. Among these, Class I PI3Ks, which differentiated into IA and IB subclasses, have been implicated in cancer-related processes (Fattahi et al., 2020). Upon ligation by extracellular growth signals or cytokines, PI3K is summoned to the plasma membrane, where its activation is orchestrated by receptor tyrosine kinases or G-protein coupled receptors. It catalyzes the pivotal transformation of phosphatidylinositol-4,5-bisphosphate (PIP2) to phosphatidylinositol-3,4,5-trisphosphate (PIP3) (Figure 1) (Yu et al., 2022). This conversion is a critical step that triggers the activation of Akt, a serine-threonine protein kinase endowed with pleckstrin homology (PH) domains, which are instrumental in its recruitment to the cell membrane (Zhang M. et al., 2019). Akt’s full activation is contingent upon its phosphorylation at two key residues, Thr308 by the kinase PDK1 and Ser473 by mTORC2 (Xu et al., 2020). Thus activated, Akt serves as a master regulator, orchestrating the activities of a plethora of transcription factors and signaling molecules such as forkhead box O1 (FOXO1), glycogen synthase kinase 3β (GSK3β), Bcl-2 associated agonist of cell death (Bad), and mTOR (Figure 1) (Klement et al., 2012). Akt’s inhibitory phosphorylation of pro-apoptotic targets like Bad, pro-caspase 9, and FOXO-family transcription factors is instrumental in averting apoptosis, thereby ensuring cell survival. The interplay between the Bcl-2 family member Bad and Bcl-2 is highlighted by their mutual antagonism of pro-survival signals (Figure 1) (Liu et al., 2023). mTOR is a nodal point in cellular growth, nutrient homeostasis, and metabolic regulation, with two functionally distinct mTOR complexes: mTORC1 and mTORC2. mTORC1’s activation is intricately linked to the availability of amino acids, modulated by specific amino acid sensors and the Ragulator-Rags complex that guides mTORC1 to the lysosome. Activation of mTORC1 fosters protein synthesis, nucleotide generation, and curbs autophagy and proteasomal degradation pathways (Yu et al., 2022). mTORC2, functioning upstream in the pathway, phosphorylates and thereby activates Akt on Ser473 (Klement et al., 2012) (Figure 1). Under homeostatic conditions, the PI3K/Akt/mTOR pathway is a guardian of cellular integrity and function. However, its dysregulation can be a harbinger of malignancy, fostering unchecked cellular proliferation and growth. The insidious nature of such dysregulation often underpins the resistance of cancer cells to chemotherapy, heightening therapeutic challenges and fostering the recurrence and aggressive progression of disease (Alharbi et al., 2023) (Figure 1).

## 3 The role of PI3K/Akt/mTOR pathway in cancer therapeutics

The PI3K/Akt/mTOR signaling cascade is a critical regulatory hub that is frequently hyperactivated or dysregulated across a spectrum of human cancers, exerting control over a plethora of cellular processes pivotal to tumor biology, such as cell survival, proliferation, growth, metabolic regulation, angiogenesis, and metastasis (Su et al., 2023). This pathway is responsive to a multitude of stimuli, including extracellular growth signals and intracellular metabolic and nutritional cues, with its dysregulation potentially leading to a variety of pathophysiological conditions (Vidal et al., 2022). The aberrant upregulation of PI3K/Akt/mTOR signaling is a defining characteristic of many cancers and is intimately associated with the neoplastic transformation and tumor progression (Zhang M. et al., 2019). The genesis of oncogenic signals emanating from an overactive PI3K is a well-documented phenomenon (Vogt and Hart, 2011). The production of PIP3 serves as a docking site for Akt and 3-phosphoinositide-dependent protein kinase-1 (PDK1), facilitating their accumulation at the plasma membrane. Post-activation, Akt translocates from the membrane to the cytoplasm and nucleus, where it engages with a multitude of substrates (Glaviano et al., 2023). Akt functions as a key modulator in the metabolic reprogramming of cancer cells, particularly in glycolysis and oxidative phosphorylation, a role that is executed through diverse mechanisms, including the regulation of hexokinase expression (Nepstad et al., 2019). The overactivation of Akt has been consistently implicated in a range of malignancies, spanning from non-small-cell lung cancer to prostate, colorectal, renal, and germ cell tumors (Jin et al., 2020) (Figure 1).

The PI3K/Akt/mTOR signaling pathway has emerged as a promising target for cancer therapeutics, offering a compelling avenue for intervention against malignancy (Pearson et al., 2024). Strategies aimed at curtailing the hyperactivity of the PI3K/Akt/mTOR signaling pathway encompass the use of PI3K inhibitors, Akt inhibitors, and mTOR inhibitors (Ellis and Ma, 2019; Dubey et al., 2024). PI3K inhibitors can exert their effects by augmenting DNA damage, inhibiting BRCA1/2 function, and disrupting homologous recombination repair, thereby dampening the downstream signaling emanating from this pathway (Ibrahim et al., 2012; Rehman et al., 2012). Several PI3K inhibitors, namely, Idelalisib (Raedler, 2015), Copanlisib (Elmenier et al., 2019), Duvelisib (Rodrigues et al., 2019) and Alpelisib (Markham, 2019), have received regulatory approval for the treatment of various malignancies. Akt inhibitors are designed to specifically impede Akt kinase activity, effectively thwarting the activation of mTORC1 and attenuating the downstream consequences of aberrant PI3K signaling. GSK2141795, a pan-Akt kinase inhibitor administered orally, has demonstrated safety, tolerability, and pharmacodynamic efficacy in Phase I clinical trials, with observed decreases in tumor size and a median duration of stable disease of over 6 months in patients exhibiting an activated PI3K pathway (Aghajanian et al., 2018). mTOR inhibitors, such as the Halitulin analog ICSN3250, act by competing with and replacing phospholipids in the FRB domain of mTOR. LY3023414 is another mTOR inhibitor that has shown to suppress the PI3K/mTOR/DNA-PK pathway *in vitro*, exerting an antitumor effect (Zou et al., 2020). In a Phase II trial, Taylor et al. evaluated the combination of the mTOR inhibitor everolimus and the anti-angiogenic agent bevacizumab in ovarian, peritoneal, and fallopian tube cancers. Among 50 patients, 14 remained progression-free at 6 months, with most experiencing stable disease but also severe toxicities (Grade 3–4) such as hypertension (Taylor et al., 2020).

## 4 Cardiotoxic effects of the PI3K/Akt/mTOR pathway inhibitor

Cardiotoxicity, a critical side effect of anticancer therapeutics, demands meticulous attention in the field of oncology. As advancements in cancer treatment extend the lifespan of patients, the prevalence and impact of drug-induced cardiotoxicity have correspondingly risen (Liu and Li, 2024). The PI3K/Akt/mTOR signaling pathway, with its extensive involvement in cellular homeostasis, is integral to a multitude of physiological processes. Disruptions within this pathway have been linked to a spectrum of human pathologies, with cardiovascular diseases being notably affected (Fruman et al., 2017; Sadasivan et al., 2020) (Figure 1). Notably, proper activation of this pathway is recognized to confer protective effects against atherosclerosis. Specifically, the engagement of mitochondrial PI3K/Akt signaling components with endothelial nitric oxide synthase (eNOS) has been observed to enhance endothelial function, thereby mitigating atherosclerotic progression (Ji et al., 2022). Disruptions in the PI3K/Akt/mTOR signaling pathway have been implicated in the etiology of cardiac dysfunction, electrical remodeling, and vascular injury, culminating in the pathogenesis of cardiovascular disease (Sadasivan et al., 2020). Consequently, therapeutic strategies that inhibit this pathway must be judiciously evaluated, considering the potential trade-offs between oncological benefits and cardiovascular safety. The balance between cancer treatment efficacy and the preservation of cardiac health is a nuanced challenge that requires a concerted research effort and clinical vigilance.

## 5 Botanical drug metabolites with bidirectional effects on the PI3K/Akt/mTOR pathway

The aforementioned research indicates that the PI3K/Akt/mTOR pathway remains stable under homeostatic conditions, and disturbances, whether from excessive activation or insufficient activation, can lead to disease. To identify botanical drug metabolites that exert bidirectional regulatory effects on the PI3K/Akt/mTOR pathway—thereby providing cardioprotective and antitumor benefits—we conducted a search in PubMed using the keywords: “PI3K/Akt,” “Natural product,” “botanical drug,” “metabolite,” “Monomer,” “Alkaloid,” “Organic acid,” “Phenylpropanoid,” “Quinone,” “Flavonoid,” “Terpenoid,” “Triterpenoid saponin,” “Steroidal saponin,” “Cardenolide,” “Tannin,” “Cardioprotective,” “Cancer,” and “Tumor.” The search strategy is detailed in the Supplementary Material. Inclusion Criteria: (1) Mechanism involves the PI3K/Akt/mTOR pathway. (2) Botanical drug metabolites that exhibit both antitumor and cardioprotective effects. Exclusion Criteria: (1) Only literature on botanical drug metabolites with antitumor effects can be retrieved, or only literature on botanical drug metabolites with cardioprotective effects can be retrieved. (2) Metabolites that demonstrate both antitumor and cardioprotective effects but do so through mechanisms unrelated to the PI3K/Akt/mTOR pathway. (3)Research that did not involve botanical drug metabolites. Through this search, we identified eight potential botanical drug metabolites, and further literature review revealed that six of these metabolites indeed possess bidirectional regulatory effects on the PI3K/Akt/mTOR pathway. These metabolites are Ruscogenin, Sulforaphane, Naringenin, Kaempferol, Poncirin, and Puerarin.

Ruscogenin (C_27_H_42_O_4_), a steroid saponin extract from the dried rhizomes of the Liliaceae plant *Ophiopogon japonicus (L.f.) Ker-Gawl.* (also named *O. japonicus (Thunb.) Ker Gawl.*). Study has shown that Ruscogenin showed pro-apoptotic and anti-metastatic effects, can reduce the phosphorylation of Akt, mTOR, and p70S6K in a dose-dependent manner in prostate cancer cells (Wang et al., 2018). In human hepatoma cell lines, Ruscogenin inhibits Akt/mTOR phosphorylation, significantly reduces HIF-1α levels, and effectively suppresses cancer cell migration, invasion, and lung metastasis formation (Hua et al., 2018). The vascular endothelium plays a pivotal role in maintaining vascular permeability, which is fundamental to vascular health. Endothelial dysfunction is a key feature of numerous cardiovascular pathologies (Wu et al., 2024). Study has investigated the mechanisms by which Ruscogenin affects endothelial cell (EC) apoptosis. The results indicated that Ruscogenin has a dose-dependent effect on increasing Akt phosphorylation, and treatment with a PI3K inhibitor blocks this phosphorylation. Ruscogenin could enhance the survival of ECs and therefore has potential clinical use in the treatment of cardiovascular diseases (Qiu et al., 2014). Furthermore, Ruscogenin has demonstrated the capacity to modulate endothelial permeability by attenuating the phosphorylation of Src, PI3K, and Akt in human umbilical vein endothelial cells exposed to tumor necrosis factor-alpha (TNF-α), a process that depends on Src activity. Ruscogenin can significantly ameliorate TNF-α-induced vascular endothelial hyperpermeability by modulating the Src/PI3K/Akt pathway (Zhang et al., 2017). Studies have also shown that Ruscogenin inhibits Caspase-3 activity in myocardial ischemia/reperfusion (MI/R) mice, exerting cardioprotective effects without significantly affecting Akt phosphorylation levels (Yang et al., 2021) (Figure 1; Table 1). Sulforaphane (C_6_H_11_NOS_2_), an organosulfur compound classified within the isothiocyanate group, primarily occurring naturally in cruciferous vegetables. Sulforaphane has been shown to enhance the phosphorylation levels of Akt in cardiomyocytes when treated with 5 µM Sulforaphane. This activation of the PI3K pathway leads to a time-dependent upregulation of antioxidant/detoxification enzymes, such as glutathione reductase and glutathione S-transferase, at both transcriptional and translational levels (Leoncini et al., 2011). In rats subjected to ischemia/reperfusion, the administration of broccoli extract rich in sulforaphane increased Akt phosphorylation levels, mitigating ischemia/reperfusion injury (Mukherjee et al., 2010). Furthermore, when Sulforaphane was used in combination with acetazolamide, it targets the PI3K/Akt/mTOR pathway for the treatment of bladder cancer. Sulforaphane alone inhibited partial phosphorylation of Akt and resulted in a reduction of p-mTOR (Islam et al., 2016). In lung bronchial carcinoid (BC) xenografts model, Sulforaphane can also notably reduced the ratios of phosphorylated Akt to total Akt (p-Akt/Akt) and phosphorylated mTOR to total mTOR (p-mTOR/mTOR), and inhibited PI3K expression (Mokhtari et al., 2021) (Figure 1; Table 1). Naringenin (C_15_H_12_O_5_), a flavonoid classified under the flavanones subcategory, is found in various citrus fruits, bergamot, tomatoes, and primarily in its glycoside form, naringin. Studies have shown that 200 μM naringenin likely stimulates *LDLr* gene expression by increasing the phosphorylation of PI3K, which in turn enhances the mRNA levels of the transcription factors SREBP-1a and SREBP-2. When PI3K inhibitors are used, the stimulated SREBP-1a promoter activity is reduced. Given that elevated plasma concentrations of low-density lipoprotein cholesterol (LDL-c) significantly contribute to the incidence of atherosclerosis and coronary artery diseases, naringenin may exert cardioprotective effects by activating the PI3K/Akt/mTOR pathway to downregulate LDLr expression (Bawazeer et al., 2017). Study showed that Naringenin induces PI3K/Akt signaling transduction pathways in prostate cancer cells to affect proliferation, migration, and apoptosis. The phosphorylation of Akt by Naringenin can be inhibited by PI3K inhibitor in prostate cancer cells (Lim et al., 2017) (Figure 1; Table 1). Kaempferol (C_15_H_10_O_6_), a natural flavonoid, occurs abundantly in fruits and vegetables. Kaempferol treatment inhibited the expression of phosphorylated PI3K (p-PI3K), phosphorylated Akt (p-Akt), and phosphorylated mTOR (p-mTOR) which induces apoptosis and autophagy in human cervical cancer cells (Kashafi et al., 2017; Choi et al., 2024). Research indicated that Kaempferol can increase the phosphorylation levels of Akt in streptozotocin-induced male diabetic rats. This suggests that the cardioprotective effect of Kaempferol is enhanced through the inhibition of apoptosis by blocking the phosphorylation of the Akt/glycogen synthase kinase (GSK)-3β and p38 mitogen-activated protein kinase/extracellular signal-regulated kinase signaling pathways in heart failure-induced diabetic rats (Zhang et al., 2019a) (Figure 1; Table 1). Poncirin (C_28_H_34_O_14_), a flavanone glycoside with a bitter taste, is extracted from the dried immature fruit of *Citrus trifoliata L.*, belonging to the Rutaceae family. Poncirin exhibits dual regulatory effects on the PI3K/Akt signaling pathway depending on pathological contexts. In cancer models, studies demonstrate that poncirin administration significantly downregulates p-PI3K and p-Akt expression levels in both cisplatin-resistant osteosarcoma and breast cancer cell lines, suggesting its antitumor potential through PI3K/Akt pathway suppression (Zhao et al., 2021; Yun et al., 2024). Intriguingly, this compound shows paradoxical cardioprotective effects under ischemic conditions. Pretreatment with poncirin markedly activates the PI3K/Akt pathway in both anoxia-reoxygenation and ischemia-reperfusion injury models, as evidenced by enhanced phosphorylation levels of key signaling molecules. Importantly, these protective effects were completely abolished by co-administration of PI3K inhibitors, confirming the pathway-dependent mechanism (Li et al., 2022) (Figure 1; Table 1). Puerarin (C_21_H_20_O_9_), a phytoestrogen derived from the root of *Pueraria lobata (Willd.) Ohwi* (also named *Pueraria montana* var. *lobata (Willd.) Maesen and S.M. Almeida ex Sanjappa & Predeep*), can reverse the LPS-mediated downregulation of Akt activation and upregulate the expressio of P-Akt in Rat H9c2 cardiomyocytes, inhibit the expression of the apoptotic factor Caspase-3. However, the cardioprotective effects of Puerarin are abolished by inhibitors of Akt and PI3K (Li et al., 2017; Yen et al., 2023). Puerarin can inhibit the phosphorylation of mTOR and Akt in pancreatic cancer cells, but its inhibitory effect is eliminated by mTOR activators. Additionally, Puerarin binds to the kinase domain of the mTOR protein, affecting the activity of the surrounding amino acid residues associated with the binding of the ATP-Mg^2+^ complex (Zhu et al., 2021). In addition to its effects on pancreatic cancer cells, Puerarin also demonstrates the capacity to inhibit Akt phosphorylation in human lung adenocarcinoma cell lines (Hu et al., 2018) (Figure 1; Table 1).

**TABLE 1:** Botanical drug metabolites with bidirectional effects on the PI3K/Akt/mTOR pathway.

Metabolite of botanical drug	Model	Dose range	Minimal active concentration	Experimental group	Controls	Duration	Action	References
Ruscogenin	Human prostate cancer cell lines	0, 2.5, 5, 10 µM	2.5 µM	Ruscogen; Ruscogenin + PI3K inhibitor	PI3K inhibitor; Normal control	48 h	P-Akt↓, P-mTOR↓, P-PDK1↓	Wang et al. (2018)
Ruscogenin	Human hepatoma cell lines	10, 20, 40, 50, 100 µM	10 µM	Ruscogen; Ruscogenin + Akt inhibitor	Akt inhibitor; Vehicle	24 h	P-Akt↓, P-mTOR↓, HIF-1α↓	Hua et al. (2018)
Ruscogenin	Human umbilical vein endothelial cells	0, 1, 2, 5 µM	5 µM	Ruscogen; Ruscogenin + PI3K inhibitor	PI3K inhibitor; Normal control	24 h	P-Akt↑	Qiu et al. (2014)
Ruscogenin	MI/R injury model	0.75 mg/kg	0.75 mg/kg	Ruscogenin	Sham group; Model group	24 h	Caspase-3↓	Yang et al. (2021)
Sulforaphane	Bladder tumor cells	2.5, 5, 10, 20, 40, 80 µM	10 µM	Sulforaphane; Sulforaphane + PI3K/mTOR inhibitor	Normal control; PI3K/mTOR inhibitor	7 days	P-Akt↓, P-mTOR↓	Islam et al. (2016)
Sulforaphane	Lung bronchial carcinoid xenografts	40 mg/kg	40 mg/kg	Sulforaphane	Normal control	14 days	p-Akt/Akt↓, p-mTOR/mTOR↓, PI3K↓	Mokhtari et al. (2021)
Sulforaphane	Cardiomyocytes	5 µM	5 µM	Sulforaphane; Sulforaphane + PI3K inhibitor	Normal control; PI3K inhibitor	24 h	P-Akt↑	Leoncini et al. (2011)
Naringenin	AOM/DSS induced orthotopic colorectal cancer model	20, 40 mg/kg	40 mg/kg	Naringenin	Control, AOM/DSS model	11 weeks	P-mTOR↓	Wang et al. (2024)
Naringenin	Prostate cancer cells	0, 5, 10, 20, 50 100 µM	50 µM	Naringenin; Naringenin + PI3K inhibitor	Normal control; PI3K inhibitor	48 h	P-Akt↓	Lim et al. (2017)
Naringenin	Cardiac hypertrophy model: aortic banding surgery model	100 mg/kg	100 mg/kg	Naringenin; Naringenin + Aortic banding surgery model	Normal control; Aortic banding surgery model	8 weeks	P-Akt↓	Zhang et al. (2015)
Naringenin	Human hepatoma HepG2 cell line	25–200 µM	100 µM	Naringenin	Negative control	4, 24 h	P-PI3K↑	Bawazeer et al. (2017)
Kaempferol	Cervical cancer cells	0, 20, 40, 60, 80, 100 µM	40 µM	Kaempferol; Kaempferol + PI3K inhibitor	Normal control; PI3K inhibitor	24, 48, 72 h	P-PI3K↓, P-Akt↓, P-mTOR↓	Choi et al. (2024)
Kaempferol	Cervical cancer cells	25–100 µM	100 µM	Kaempferol	Human Foreskin Fibroblast cells	72 h	PI3K↓, AKT↓	Kashafi et al. (2017)
Kaempferol	Heart failure model: Streptozotocin-induced male diabetic rats	10 mg/kg, 20 mg/kg	10 mg/kg	Diabetic + isoproterenol + Kaempferol	Diabetic; diabetic + isoproterenol	41, 42 days	P-Akt↑	Zhang et al. (2019a)
Poncirin	Cisplatin-resistant osteosarcoma cells	40, 80 µM	40 µM	Poncirin	Negative control	48 h	P-PI3K↓, P-Akt↓	Zhao et al. (2021)
Poncirin	Breast cancer cell line	4, 8, 16 µM	4 µM	Poncirin	Normal control	24 h	P-PI3K↓, P-Akt↓	Yun et al. (2024)
Poncirin	*In vivo*: Ischemia-reperfusion injury model	*In vivo*: 0–120 mg/kg/d	*In vivo*: 30 mg/kg	*In vivo*: Ischemia-reperfusion + Poncirin; ischemia-reperfusion + Poncirin + PI3K inhibitor	*In vivo*: Control; ischemia-reperfusion	*In vivo*: 3 days	P-Akt↑	Li et al. (2022)
	*In vitro*: Anoxia-reoxygenation model	*In vitro*: 12.5, 25, 50, 100, 150 µM	*In vitro*: 20 µM	*In vitro*: Anoxia-reoxygenation + Poncirin; Anoxia-reoxygenation + Poncirin + PI3K inhibitor	*In vitro*: Control; Anoxia-reoxygenation	*In vitro*: 24 h		
Puerarin	Human lung adenocarcinoma cell lines	5, 10, 20 µM	10 µM	Puerarin	Normal control; PI3K inhibitor	72 h	P-Akt↓	Hu et al. (2018)
Puerarin	*In vivo*: Nude mouse xenograft model	*In vivo*: 50 mg/kg	*In vivo*: 50 mg/kg	*In vivo*: Puerarin; Puerarin + mTOR activator	*In vivo*: Normal control; Pancreatic cancer cells; mTOR activator	*In vivo*: 1 month	P-mTOR↓, P-Akt↓	Zhu et al. (2021)
	*In vitro*: Pancreatic cancer cells	*In vitro*: 0.2, 0.5 mM	*In vitro*: 0.2 mM	*In vitro*: Puerarin	*In vitro*: Normal control; Pancreatic cancer cells	*In vitro*: 24 h		
Puerarin	H9c2 cells with lipopolysaccharide or H2O2 stimulation	0, 20, 40 µM	20 µM	Lipopolysaccharide + Puerarin; lipopolysaccharide + Puerarin + Akt inhibitors	Normal control; Lipopolysaccharide; Akt inhibitors	24 h	P-Akt↑	Yen et al. (2023)
Puerarin	Rat H9c2 cardiomyocytes	1, 10, 100 μg/mL	100 μg/mL	Puerarin	Normal control; PI3K inhibitor; daunorubicin	48 h	P-Akt↑, Caspase-3↓	Li et al. (2017)

Note: MI/R, ischemia/reperfusion; AOM/DSS, Azoxymethane/dextran sodium sulfate.

In the included literature, Akt is identified as a key target with bidirectional regulatory effects. It undergoes critical post-translational modifications, with phosphorylation being essential for its activation. The Akt protein has two primary phosphorylation sites that vary slightly among its three isoforms (Akt1, Akt2, and Akt3). These sites are a threonine residue within the kinase domain (Akt1 at position 308, Akt2 at 309, and Akt3 at 305) and a serine residue in the hydrophobic motif (Akt1 at 473, Akt2 at 474, and Akt3 at 472) (Abeyrathna and Su, 2015). Among these, the phosphorylation of Thr308 and Ser473 stands out as particularly influential for Akt functionality. The kinase PDK1 is identified as the direct upstream regulator of Thr308 phosphorylation, whereas the mTORC2 predominantly orchestrates Ser473 phosphorylation (Dirimanov and Högger, 2019) (Figure 1).

This study analyzed the mechanisms by which botanical drug metabolites exert anti-tumor and cardiovascular protective effects through the PI3K/Akt/mTOR pathway. It provided a reference for the study of the bidirectional regulatory effects of botanical drug metabolites in the PI3K/Akt/mTOR pathway and for reducing the cardiotoxic side effects of anti-tumor drugs. In future research, the reasons for the bidirectional regulatory mechanisms can be further analyzed from the perspective of the phosphorylation mechanisms of the PI3K/Akt/mTOR pathway, and attention should also be paid to the selective differences of botanical drug metabolites under different body mechanism conditions and the impact of cellular differences on the mechanisms of botanical drug metabolites. However, this study also has the following shortcomings: (1) The number of studies included was small, and the conclusions lacked sufficient supporting evidence. (2) The experimental design of the study itself was not rigorous enough. For example, none of the studies designed a method for the extraction of botanical drug metabolites. *In vitro* experiments conducted by Nora A. Bawazeer (Bawazeer et al., 2017), Elham Kashafi (Kashafi et al., 2017), Hao Yun (Yun et al., 2024), and Liujing Zhao (Zhao et al., 2021) only included comparisons against normal controls and did not involve the use of PI3K/Akt/mTOR pathway inhibitors. (3) The bidirectional mechanisms were not from the same study but were integrated conclusions from different studies, which could lead to errors in the conclusions. (4) The authenticity of the conclusions of the included studies also needs further verification. (5) The study conclusions were only limited to the expression levels of P-PI3K and P-Akt, and could not provide references on multiple aspects of the mechanisms, such as the impact of molecular microscopic mechanisms and changes in phosphorylation sites on the results, which also led to certain limitations in the reference significance of the results themselves.

## 6 Conclusion

Given the distinct mechanisms by which the PI3K/Akt/mTOR signaling pathway operates in tumors *versus* cardiovascular diseases, and the fact that multiple botanical drug metabolites have demonstrated the ability to exert bidirectional regulatory effects on different diseases through this pathway, this study takes the PI3K/Akt/mTOR pathway as an entry point to investigate the bidirectional regulatory effects of botanical drug metabolites within both oncology and cardiology. The overactivation of the PI3K/Akt/mTOR pathway is a key driver of tumorigenesis and is widely recognized as a hallmark of various human malignancies. Conversely, this pathway’s activation has also been linked to protective effects against atherosclerosis. The divergent functional roles of this pathway in cancer and cardiovascular medicine may elucidate the cardiotoxicity observed with PI3K/Akt/mTOR inhibitors in cancer treatments. To mitigate disease risk, it is imperative that the PI3K/Akt/mTOR pathway is maintained within an optimal range; disruptions - whether from overactivation or underactivity - can precipitate disease progression. When the pathway is overactivated, Ruscogenin, Sulforaphane, Naringenin, Kaempferol, Poncirin, and Puerarin are worth studying as they may exert anticancer effects by inhibiting the phosphorylation levels of the PI3K/Akt/mTOR pathway. Conversely, when expression of the PI3K/Akt/mTOR pathway is insufficient, interestingly, these metabolites may also provide cardioprotective effects by activating the phosphorylation levels of the pathway. The phosphorylation levels and sites within the PI3K/Akt/mTOR pathway and the selectivity of botanical drug metabolites for cellular subtypes may offer new insights for developing therapeutic strategies aimed at treating malignancies while minimizing the risk of cardiotoxicity. Although the findings indicate that botanical drug metabolites exert bidirectional regulatory effects on the PI3K/Akt/mTOR pathway, the number of studies included is limited, and some study designs exhibit flaws. Moreover, the anticancer and cardioprotective mechanisms are grounded in separate lines of research. Therefore, further high-quality experimental validation is required, alongside more in-depth mechanistic analyses, with a focus on systematically investigating the bidirectional regulatory mechanisms of botanical drug metabolites, as well as conducting additional clinical trials for validation.
