# Supplementary material for: Potential bidirectional regulatory effects of botanical drug metabolites on tumors and cardiovascular diseases based on the PI3K/Akt/mTOR pathway
Source: Front Pharmacol. 2025 Mar 24;16:1467894. doi: 10.3389/fphar.2025.1467894 (PMC11973345; doi:10.3389/fphar.2025.1467894)
Supplement: Supplementary file 1 [file DataSheet1.docx]

Supplementary Material

# Search String

Search String Used:

(((PI3K/AKT[Title/Abstract]) AND (Natural product[Title/Abstract])) AND (tumor[Title/Abstract])) AND (cardioprotective[Title/Abstract])

Total Articles Retrieved: 1

Search String Used:

(((PI3K/AKT[Title/Abstract]) AND (monomer[Title/Abstract])) AND (cancer[Title/Abstract])) AND (cardioprotective[Title/Abstract])

Total Articles Retrieved: 1

Search String Used:

(((PI3K/AKT[Title/Abstract]) AND (monomer[Title/Abstract])) AND (tumor[Title/Abstract])) AND (cardioprotective[Title/Abstract])

Total Articles Retrieved: 1

Search String Used:

(((PI3K/AKT[Title/Abstract]) AND (Quinone[Title/Abstract])) AND (cancer[Title/Abstract])) AND (cardioprotective[Title/Abstract])

Total Articles Retrieved: 1

Search String Used:

(((PI3K/AKT[Title/Abstract]) AND (Quinone[Title/Abstract])) AND (cancer[Title/Abstract])) AND (cardioprotective[Title/Abstract])

Total Articles Retrieved: 1

Search String Used:

(((PI3K/AKT[Title/Abstract]) AND (Flavonoid[Title/Abstract])) AND (tumor[Title/Abstract])) AND (cardioprotective[Title/Abstract])

Total Articles Retrieved: 2

Search String Used:

(((PI3K/AKT[Title/Abstract]) AND (Flavonoid[Title/Abstract])) AND (cancer[Title/Abstract])) AND (cardioprotective[Title/Abstract])

Total Articles Retrieved: 3

(((PI3K/AKT[Title/Abstract]) AND (metabolite[Title/Abstract])) AND (tumor[Title/Abstract])) AND (cardioprotective[Title/Abstract])

Total Articles Retrieved: 1

(((PI3K/AKT[Title/Abstract]) AND (Metabolite[Title/Abstract])) AND (cancer[Title/Abstract])) AND (cardioprotective[Title/Abstract])

Total Articles Retrieved: 1
